# The impacts of the coronavirus pandemic on the mental health of Brazilian diagnosed with COVID-19 and comparison of symptoms of depression, anxiety, insomnia, and post-traumatic stress with undiagnosed subjects

**DOI:** 10.3389/fpsyt.2023.1151253

**Published:** 2023-07-10

**Authors:** Sumayla Gabrielle Nascimento da Silva, Lucas Mendes Carvalho, Fernando Cesar de Souza Braga, Rodrigo Silveira, Ozélia Sousa Santos

**Affiliations:** ^1^Faculty of Medicine, Federal University of Pará, Altamira, Brazil; ^2^Campus ‘University City Armando de Salles Oliveira (CUASO)’, University of São Paulo, São Paulo, Brazil

**Keywords:** mental health, COVID-19, survivors, anxiety, depression

## Abstract

**Background:**

The impacts of the COVID-19 pandemic on the mental health of survivors are little known, especially regarding the occurrence of psychological disorders such as anxiety and depression. In this study, we evaluated the impacts on the mental health of Brazilian survivors who were not infected or asymptomatic with COVID-19.

**Methods:**

A cross-sectional study was conducted collecting information through an electronic form from January to May 2021. The sample consisted of 1,334 people and were divided into two groups: case, with individuals who reported a positive diagnosis of the disease, with or without symptoms, and control, who reported not being diagnosed with COVID-19 and did not present any symptoms during the collection period. Validated instruments were used to investigate symptoms of depression (Patient Health Questionnaire), anxiety (Generalized Anxiety Disorder-7), post-traumatic stress disorder (Post Traumatic Stress Disorder Checklist) and insomnia (Insomnia Severity Index). The data were presented as standard deviation or median and interquartile ranges. The chi-square test was applied for statistical significance between categorical variables, considering a *p* < 0.05.

**Results:**

Regarding post-traumatic stress levels, the case and control groups showed no differences (*p* = 0.82). The results of the research indicated that was no statistical correlation between the group that was affected by the virus infection and the group that was not affected in terms of depression (*p* = 0.9) and anxiety (*p* = 0.7). At the same time, the levels of insomnia (*p* = 0.02) demonstrated a statistical correlation between the groups. The prevalence of the analyzed mental health disorders was similar among both groups.

**Conclusion:**

In conclusion, the population of survivors of COVID-19 infection tends to show little difference in terms of developing post-traumatic stress disorder, anxiety, and depression when compared to uninfected individuals. On the other hand, disorders such as insomnia are more prevalent and show a significant difference between groups, appearing more in infected individuals.

## 1. Introduction

In December 2019, a new betacoronavirus, SARS-CoV-2 was discovered in Wuhan, China (1). The clinical manifestations are pneumonia, symptoms of fever, cough, pulmonary infiltration, dyspnea with the occurrence of myalgias, and taste and smell disorders (1).

In Brazil, the challenges brought by COVID-19 are associated with the high incidence of cases, the wide geographic distribution of the virus, and the consequent circulation of variants. The general picture of the disease showed high mortality, resulting in efforts to access services and specialized health centers with quality of care, efficient epidemiological surveillance, and tactics to control viral spread (2).

Since the beginning of the pandemic caused by SARS-CoV-2, some studies have already shown that the social context—of mental health problems—has undergone a major change. Many self-reported cases have demonstrated a significant increase in illnesses such as depression and anxiety (3).

The perceptions of stress are individual and subjective, which means that they affect a certain group of people in different ways, even if united by a similar situation. In the case of epidemic survivors, one of the most recurrent comorbidities is related to psychiatric disorders, with an emphasis on mood disorders such as depression, anxiety, and Post Traumatic Stress Disorder (PTSD) (4).

In a study carried out by Wang et al. (5) in China with 1,200 participants, the psychological impact of COVID-19 during the first weeks in the country was analyzed. In this research, the DASS 21 scale was used to measure the levels of depression, stress, and anxiety in the volunteers. The results showed that 651 research volunteers (53.8%) reported moderate or severe psychological impact, compared to 24.5% who reported minimal psychological impact. Still, 16.5% were considered, through the score, with moderate, severe, or extremely severe depression. Furthermore, 28.8% attested to moderate or severe anxiety and 32.2% to some level of stress.

Moreover, regarding changes in sleep quality during the COVID-19 pandemic, Barros et al. (6) in their study with data from “ConVid—Research of Behaviors,” which was developed by Fundação Oswaldo Cruz, analyzed that 37.1% of male volunteers started to have sleep problems during the pandemic, while this number was of 49.8% in women. In addition, a greater number of women showed worsening previous sleep problems during the pandemic.

The direct—or indirect—relationship between COVID-19 infection, during the pandemic, and psychiatric disorders is a link that demonstrates the varied consequences that such periods can cause in individuals of a population. A pandemic not only brings effects related to physical health or related to the pathophysiology of a particular virus or bacteria, but also a chain of social, cultural, and economic repercussions that significantly interfere with the increase in the occurrence of disorders such as depression, anxiety, post-traumatic stress, and insomnia (4). The increase in these disorders, nowadays, also means an increase in medication dependence, stigmatization, and a decrease in the quality of life of these individuals in various social spheres such as family, friends, and work, which harm—individually or collectively—an entire feedback system that generates more psychic suffering and non-psychiatric illness, as well as the modification of the socio-environmental context and its health determinants. In addition, the COVID-19 pandemic and all its consequences showed a complete picture of how institutions and public policies can act in the event of pandemics and epidemics in the future, given the possibility of new episodes occurring in this century (1).

Although many studies have investigated the impact of the COVID-19 pandemic on the mental health of different social groups around the world, few studies have demonstrated the impacts on the mental health of patients who survived the pandemic. For this reason, it is still urgent that more work be carried out to analyze the previous impacts, from short to long term, on the total panorama of the Brazilian population, which was—and continues—extremely affected by the biological, social, and economic pandemic’s consequences. Brazil is a large country with a vast diversity of regions that differ in culture, socioeconomic conditions, and healthcare resources (7). Hence, this study aims to assess the association between the coronavirus infection experience the mental health among people who survived COVID-19 infection in different regions of Brazil in terms of depression, anxiety, insomnia, and post-traumatic stress disorder.

## 2. Methods

This is a cross-sectional study conducted following the guidelines of the Strengthening the Reporting of Observational Studies in Epidemiology (STROBE). The study was carried out by collecting information through an electronic form from January to May 2021.

### 2.1. Sample

The sample consisted of 1,334 people over 18 years old (67.2 ± 6.7) residing in Brazilian territory. For the sample size calculation, the software G * Power 3.0.10 was used to simulate all the analyzes performed in the present study. Thus, the sample size was determined by the analysis that estimated the largest number of participants, being a chi-square test with up to 6 degrees of freedom, assuming an intermediate effect size, a significance of *p* < 0.05, and statistical power of 95%. The estimated minimum sample size was *n* = 232. However, this minimum estimated sample size was increased by 90% to ensure a better representation of the Brazilian population. Thus, based on cultural plurality in the set of 27 Brazilian states, the estimated minimum sample size increased by 186 (~80%) with an additional 22 (~10%) for possible sample loss. The inclusion criteria for this study were: currently residing in Brazilian territory; being 18 years of age or older; having or not having been diagnosed with COVID-19; and being able to answer all questions in the questionnaire coherently. The exclusion criteria included: residing outside the national territory; being under 18 years old; not answering all the questions in the questionnaire; or answering incoherently to the questions prepared. The sample consisted of individuals with specific characteristics for each group. In the case group, individuals who reported a positive diagnosis of COVID-19, with or without the manifestation of symptoms, were grouped. In the control group, individuals who reported not having been diagnosed with COVID-19 and did not present any symptoms during the collection period were grouped.

### 2.2. Procedures

**Figure 1:**
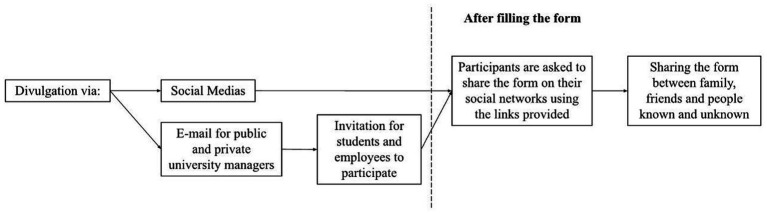
Questionnaire disclosure method.

To carry out this study, a structured questionnaire was used. To enhance the quality of the selected questions, the quality of the sample, the participant’s understanding of the selected questions, and the feasibility of the questionnaire conducted a pilot questionnaire. This questionnaire was administered to a small group of participants (*n* = 62) randomly selected from the study’s target population and sent via email. Based on the results obtained, the questions and instructions were adjusted to make clearer, more precise, and more appropriate for the sample and the study’s objective. The 62 participants of the pilot sample were not included in the final data-analysis. The final questionnaire was then administered to all study participants. It was disseminated via email and social networks. To ensure the randomization of the sample was employed a recruitment strategy that involved disseminating the study invitation through social media and targeted emails sent to professors at public and private universities. Participants were encouraged to forward the invitation to other potential participants, such as students, family, and friends (Figure 1). However, we emphasized that participation was entirely voluntary and that forwarding the invitation was not mandatory. We provided participants with feedback on the components evaluated in the questionnaire after they completed it.

The impacts of the new coronavirus pandemic on mental health were evaluated using multiple-choice questions. In addition to general demographics, the questionnaire included questions about COVID-19 treatment management, depressive symptoms, levels of anxiety and post-traumatic stress disorder (PTSD), insomnia symptoms, access to health services, previous exposure to traumatic events, stigmatization by family members, friends and/or society and coping strategies.

To accompany the research participants were offered follow-up, counseling, guidance, and specialized assistance provided by psychiatrists and medical students from the Federal University of Pará—Campus Altamira, following World Health Organization recommendations.

### 2.3. Instruments

#### 2.3.1. Assessment of depressive symptoms

Depressive symptoms were assessed using the Patient Health Questionnaire (PHQ-9) in the Portuguese language. The instrument comprises nine items, arranged on a four-point scale: 0 (not at all) to 3 (almost every day), with scores ranging from 0 to 27 to assess the frequency of signs and symptoms of depression in the last 2 weeks. A score higher than or equal to 10 is estimated as a positive indicator of major depression (8). Its original version is presented by Spitzer et al. (9) and Kroenke et al. (10), and its validation and translation in Brazil were given by Osorio et al. (11).

#### 2.3.2. Assessment of anxiety levels

The presence of anxiety symptoms was assessed using the Generalized Anxiety Disorder-7 (GAD-7) elaborated by Spitzer et al. (12) and validated by Maley (13). The translation into Portuguese was made by Pfizer (Copyright© 2005 Pfizer Inc., New York, NY). It consists of seven items, arranged on a four-point scale: 0 (never) to 3 (almost every day), with a score ranging from 0 to 21 when measuring the frequency of signs and symptoms of anxiety in the last 2 weeks.

#### 2.3.3. Assessment of post-traumatic stress levels

To assess PTSD, the Post Traumatic Stress Disorder Checklist (PCL-5) was used, which applies the criteria of the Diagnostic and Statistical Manual of Mental Disorders (DSM-5). The Brazilian version was translated by Spitzer et al. (12). It consists of 20 items arranged on a five-point scale: 0 (not at all) to 4 (extremely) to evaluate the severity of the symptoms related to traumatic experiences.

#### 2.3.4. Assessment of insomnia symptoms

The Insomnia Severity Index (ISI) was validated by Bastien et al. (14) and its validation was revised by Buysse et al. (15). The ISI was used in Portuguese language and consists of five items, ranging from 0 to 7 for no clinically significant insomnia to 22–28 for severe insomnia.

#### 2.3.5. Assessment of clinical progression scale of COVID-19

The WHO Clinical Progression Scale of COVID-19 was used as a method to divide the groups diagnosed (case) and undiagnosed (control) with COVID-19. This scale ranges from 0 to 10, with 0 representing uninfected individuals with no viral RNA detected, therefore, for undiagnosed individuals, the range from 1 to 9 represents the subjects who received a diagnosis of COVID-19. The higher the score on this scale, the greater the severity of the symptoms presented by the participants, and 10 represents those who have died from the disease (16).

### 2.4. Data analysis

Continuous data were presented as standard deviation or median and interquartile ranges, depending on distributions, and categorical as percentages. The Pearson or Spearman correlation test was used (in the case of asymmetric distribution). For the test on categorical variables, Pearson’s chi-square test was applied with the correction of Fisher’s exact test when there were < 6 participants in a category. In case of statistical significance, the adjusted residual values >2 were analyzed to identify which categories are influencing *p*-values.

To analyze the magnitude of the differences between the groups, the effect sizes were observed using Phi (Φ), in 2 × 2 tables and Cramer’s V, in tables above 2 × 2, assuming values of “Null or Very Weak” for ranges between 0 and 0.05, “Weak” for ranges between 0.05 and 0.10, “Moderate” if between 0.10 and 0.15, “Strong” for values above 0.15–0.25 and “Very Strong” for values above 0.25 (17).

For all tests, a value of *p* < 0.05 was adopted as an indication of significance. All statistical analyses were processed in SPSS software (Statistical Package for Social Sciences), version 23.0.

### 2.5. Ethical-legal aspects

The research project was submitted to the Human Research Ethics Committee for approval through registration on Plataforma Brazil. Participants were informed about the objectives of the study, the voluntary nature of participating, and the need to sign the Free and Informed Consent Term as recommended by Resolutions 466/2012 and 510/2016 of the National Health Council. Data collection was performed after approval by the Ethics Committee of the Institute of Health Sciences of the Federal University of Pará with the following CAAE number: 36046620.0.0000.0018. All subjects provided electronically informed consent before enrollment. The informed consent page presented two options (I accept/I do not accept). Only subjects who chose the “accepted” option advanced to the electronic questionnaire, and subjects could interrupt the process at any time.

## 3. Results

**Figure 2:**
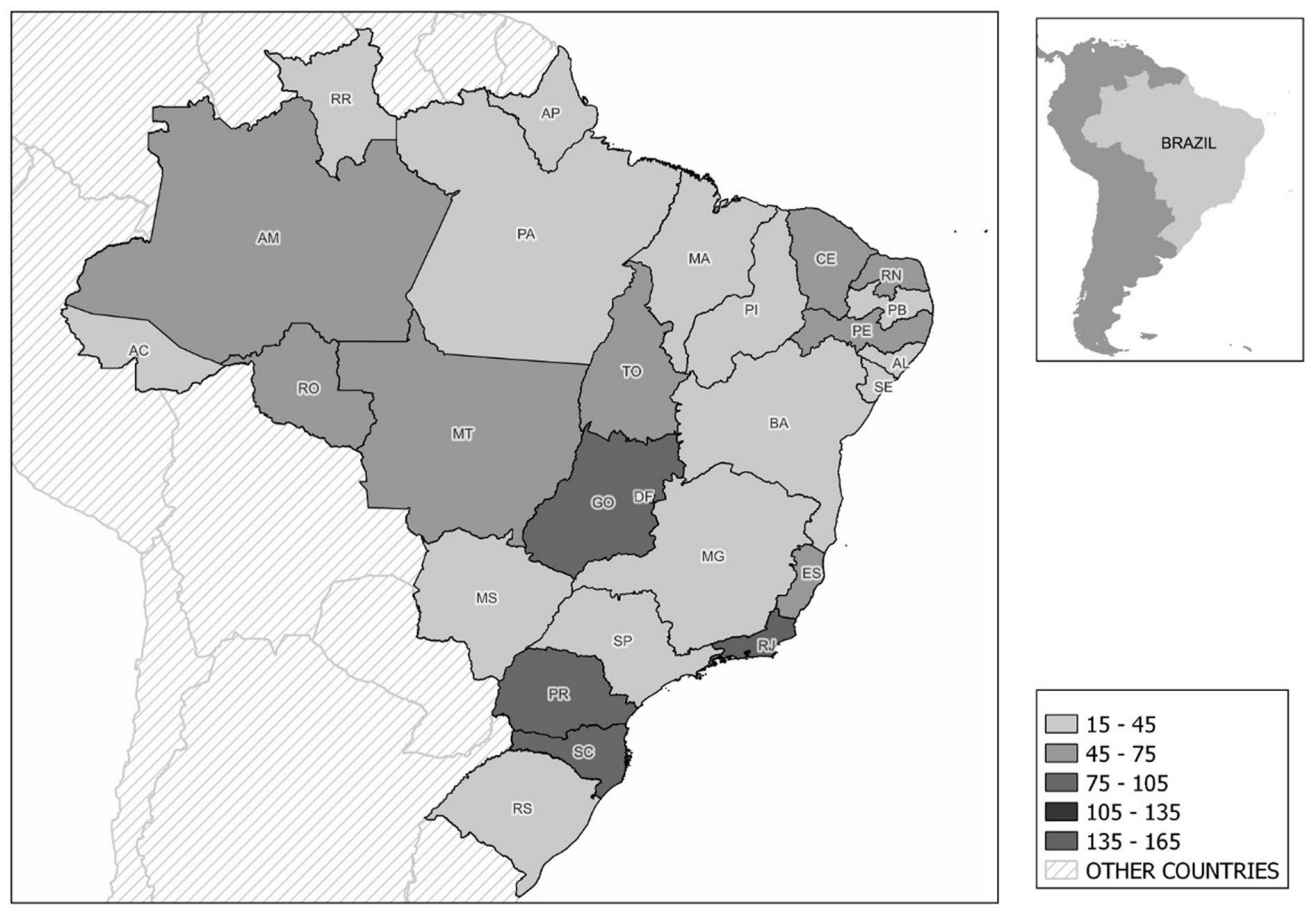
Sample distribution by Brazilian states.

The research participants totaled 1,334 people, with 668 individuals from the case group, corresponding to those who were diagnosed with the coronavirus, and 666 from the control group. There were participants from all 27 Brazilian states with distribution ranging from *n* = 15 in Amapá and Acre to *n* = 165 in Rio de Janeiro (Figure 1). Additionally, 62 people participated in the pilot study. In the study, 70.6% of respondents were female and 28.4% were male (*n* = 1,334) (Table 1 and Figure 2).

**Table 1:** Sociodemographic characteristics of the research participants.

	Case (*n* = 668)	Control (*n* = 666)	Effect size	*P*	Power (1 − β)
	*F* = 71.7%	*F* = 69.7%			
Gender			Φ = 0.02	0.43	0.142
	*M* = 28.3%	*M* = 30.3%			
Age	34 (18–72)	36 (18–75)	Φ = 0.08^†^	<0.001	0.49
Marital status					
Married	45.2%	47.0%			
Divorced	5.5%	6.3%	Φ = 0.05^†^	0.3	0.71
Single	47.9%	46.2%			
Widower	1.3%	0.5%			
Education					
Elementary school	0.6%	0.0%			
High school	9.9%	6.6%			
University education	29.3%	24.6%	Φ = 0.106^††^	0.004	0.63
Technical education	2.4%	2.9%			
Postgraduate studies	57.8%	65.5%			
Religion					
Atheist or agnostic	13.5%	25.1%			
Buddhist	0.3%	0.6%			
Catholic or protestant	64.7%	49.5%	Φ = 0.176^††^	0.176	0.98
Spiritist	10.5%	10.5%			
Jewish	0.3%	0.3%			
African origin	1.2%	2.4%			
Others	9.6%	11.6%			

F, female; M, male. ^†^Small effect size; ^††^moderate effect size. Researchers’ collection.

The median (50th quartile) of participants’ age was 34 years for the case group and 36 years for the control group. As for marital status, 615 (46.1%) of respondents declared themselves to be married and 628 (47.1%) were single. Widowed and divorced totaled 91 (6.7%). Regarding the level of education, 4 (0.3%) declared having completed or incomplete elementary education; 110 (8.2%) said they had completed or incomplete high school; 360 (27%) said they had university education; 35 (2.6%) confirmed having completed technical education and 825 (61.8%) reported being or having completed postgraduate studies.

As for the religion surveyed among respondents, 257 (19.3%) said they were atheists or agnostics, 6 (0.4%) Buddhists, 762 (57.1%) Catholics or protestants, 140 (10.5%) spiritualists, 4 (0.3%) Jewish, 24 (1.8%) were of African origin and 141 (10.6%) claimed to have other religions.

It was also found that of the total sample of participants (*n* = 1,334), 559 (44.9%) reported having experienced a potentially traumatic event, where there was fear or risk of dying and that was not related to COVID-19. When asked about having witnessed traumatizing events during the new coronavirus pandemic, 378 (28.3%) answered yes, 210 (31.4%) from the case group, and 168 (25.2%) from the control group (Table 2).

**Table 2:** Participants who experienced a potentially traumatic event.

	Case (*n* = 668)	Control (*n* = 666)	Effect size	*P*	Power (1 − β)
Experienced traumatic pre-pandemic event	42.8%	47%	Φ = 0.04	0.137	0.5
Experienced traumatic events during the pandemic	31.4%	25.2%	Φ = 0.069^†^	0.01	0.5

^†^Small effect size. Researchers’ collection.

When analyzing the depression rates, through the responses received by the participants in the questionnaire (PHQ-9), it was observed that 616 (46.2%) patients showed signs of depression, with 310 (46.4%) from the group of individuals diagnosed with COVID-19 and 306 (45.9%) of the control group. Those who showed signs of anxiety totaled 390 individuals (29.2%) of the total sample, 199 (29.8%) of the SARS-CoV-2 diagnosed group, and 191 (28.7%) of the undiagnosed group. The PCL-5 checklist for analyzing Post Traumatic Stress Disorder (PTSD) showed in the study that 324 people (24.3%) of the total sample of respondents had signs of PTSD. Of these, 164 (24.6%) were in the case group and 160 (24%) were in the control group. When analyzing the signs of insomnia, it was attested that 766 people (57.4%) of the total met the criteria for the disorder, with 404 (60.5%) corresponding to the group that was diagnosed with COVID-19 and 362 (54.4%) of the group that was not diagnosed with the disease (Table 3).

**Table 3:** Depression, anxiety, PTSD, and insomnia in the sample of individuals in the case and control groups.

	Case (*n* = 668)	Control (*n* = 666)	Effect size	*P*	Power (1 − β)
Shows signs of depression	46.4%	45.9%	Φ = 0.005	0.9	0.87
Shows signs of anxiety	29.8%	28.7%	Φ = 0.01	0.7	0.68
Shows signs of post-traumatic stress disorder	24.6%	24%	Φ = 0.006	0.8	0.83
Shows signs of insomnia	60.5%	54.4%	Φ = 0.062^†^	0.03	0.5

^†^Small effect size. Researchers’ collection.

## 4. Discussion

In this study, we assessed levels of depression, anxiety, post-traumatic stress disorder, and insomnia in Brazilian survivors of COVID-19. While the impacts of COVID-19 on mental health have been widely described in various populations, survivors of COVID-19 may be more susceptible to psychological and psychiatric issues due to the impact of contracting the virus and experiencing disease symptoms (4).

When comparing the levels of depression and anxiety between subjects affected by COVID-19 (case group) and unaffected subjects (control group), we did not find a statistically significant correlation between the groups. These findings partially corroborate the results presented by Zhang et al. (18), who demonstrated an increased prevalence of anxiety in both patients infected with COVID-19 and individuals under quarantine and the general public. However, they found an increased prevalence of depression predominantly in patients who had been infected with COVID-19. Similarly, Ryal et al. (19) also demonstrated a high prevalence of psychiatric disorders in surviving patients diagnosed with COVID-19, with depression and anxiety being among the highest. Reagu et al. (20), in their study on a population in isolation and institutional quarantine in Qatar, used the same instruments as this study in similar sample size and reported that participants with positive COVID-19 PCR tests had significantly higher levels of depressive and anxiety symptoms than participants with negative tests. Other studies have linked the clinical severity of the disease to greater severity of psychiatric disorders. For example, in a study conducted in China on a general population of 432 survivors, it was found that the prevalence of anxiety disorder was 29%. However, for survivors with more severe COVID-19, the prevalence of anxiety disorder was up to four times higher than in the general population of the study (21).

Indeed, subsequent experiences from other outbreaks and epidemics have shown an increase in comorbidities among individuals who survived the diseases during the viral spread, regardless of the severity of the condition (18). The COVID-19 pandemic was no exception. It introduced a new social dynamic never before experienced by society, capable of generating emotional impacts on various segments of the population. In a recent meta-analysis focusing solely on the prevalence of psychological distress during the COVID-19 pandemic, it was found that one in three adults in the predominantly general population has anxiety or depression. Women, younger adults, individuals from lower socioeconomic backgrounds, residents in rural areas, and people with or at high risk of COVID-19 infection (suspected/confirmed cases, residents in heavily affected areas, having a history of chronic or mental conditions) were associated with higher chances of psychological distress (22). Several studies have also assessed the prevalence of mental health symptoms and disorders among healthcare professionals. Almalki et al. (23) demonstrated that over a year into the COVID-19 pandemic, the prevalence of depression, anxiety, and stress remains substantial among healthcare professionals in Saudi Arabia. Hajebi et al. (24) examined the mental health of healthcare professionals in Iran using the same instruments used in our study (PHQ-9 and GAD-7). They found that half of the participants had either generalized anxiety disorder, major depressive disorder, or both. According to Dubey and Tripathi (25), social withdrawal, isolation itself, and excessive information disseminated through social media are sufficient to increase psychological symptoms, potentially leading to anxiety, panic, and depression. Therefore, the pandemic event affects both infected and non-infected individuals in terms of psychological problems, which may justify our findings.

In addition to depression and anxiety, the prevalence of insomnia and post-traumatic stress disorder (PTSD) symptoms has been widely described in a significant proportion of COVID-19 patients (26). In our study, we found a higher prevalence of insomnia in individuals infected with COVID-19 compared to non-infected individuals. A recent meta-analysis focusing on the prevalence of depression, anxiety, and insomnia symptoms among SARS-CoV-2 infected patients revealed that sleep disorders were present in 48% of coronavirus-infected patients (26). Lin et al. (27), investigating the immediate impact of the coronavirus on subjective sleep status, evaluated over 5,000 individuals in China divided into groups ranging from those who had direct contact with the virus, such as healthcare professionals, to individuals related to the group with direct contact, such as friends and family of frontline workers. Clinical insomnia was detected in 20.05% of the subjects studied. This clearly demonstrates the correlation, as evidenced in other studies, between coronavirus infection and its impacts on sleep quality, both among individuals who had direct contact with the disease and those who, even without contracting the infection, were involved in the global social context of the pandemic.

Regarding PTSD, although the case group showed higher exposure to potentially traumatic events unrelated to COVID-19, there was no statistically significant difference in post-traumatic stress levels between the groups. In a study conducted with adults in China, the epicenter of the coronavirus pandemic, PTSD was identified as the most concerning disorder during and after the pandemic, with a prevalence of 30%. Both diagnosed and undiagnosed individuals with COVID-19 reported a higher fear of infection risk and a negative perception of the situation, leading to greater PTSD symptoms (28). These findings differ from the results of the present study, which did not find significant associations between the case and control groups for PTSD symptoms. In some other studies involving hospitalized patients diagnosed with COVID-19, the prevalence of PTSD was as high as 96.2% (29). According to certain studies, PTSD appears as a provisional diagnosis primarily in patients who were hospitalized during the COVID-19 pandemic (30). This suggests a relationship with the severity of the illness, as asymptomatic individuals who were not hospitalized did not report significant levels of PTSD.

A study conducted during the second wave of the pandemic in Iran, involving nearly 1,800 participants, showed that the prevalence of PTSD was significantly higher in hospitalized individuals and in outpatient groups receiving treatment for COVID-19 compared to the general population (31). Furthermore, a study in the United Kingdom with over 13,000 participants who were suspected or confirmed COVID-19 cases found that PTSD symptoms were disproportionately higher in patients who required hospital treatment (32). Another study by Guo et al. (33) in Mainland China observed higher levels of PTSD, with or without comorbid depression and anxiety, in COVID-19 survivor patients compared to non-infected individuals. Therefore, it is evident that several studies indicate a correlation between post-traumatic stress disorder and potentially destabilizing events on mental health, such as a pandemic.

Due to the analyses of the results of this research, the importance of studies covering this area is understood. Correlations of the disease with possible psychiatric disorders are dangerous because they make this group a risk factor for suicide and other disorders such as self-injury. Thus, individuals who contract the virus and develop the disease should be supported not only in the systemic aspects involving the primarily affected organs (such as lungs, heart, and kidneys) but also concerning their mental health regarding their internal and external suffering and the stigma created against such individuals.

However, the fact that the study design is cross-sectional does not allow for long-term follow-up of the patient to verify if there would be any changes in the profile of the patient’s involvement, mainly due to the physiological, social, and psychological sequelae resulting from the disease (34).

The results of this study are very important, as they bring to light psychological and psychiatric symptoms in their most pathological manifestations in a group of survivors of patients of COVID-19 during the pandemic period caused by this virus. This study reveals a series of precautions and alarms that the health system and health professionals must have after such a period of the COVID-19 pandemic, with the diagnosis, treatment, and life quality of the experienced population showing that the consequences of COVID were not only related to the restricted aspects of the comorbidity but also the emotional effects during this period. Based on this study, further research on these long-term psychological and psychiatric disorders in individuals who survived epidemic diseases is necessary to add more contributions and knowledge about the depth of the psychic crises of patients who survive epidemic diseases with far-reaching—like pandemics.

Although COVID-19 has not bid farewell yet, we can begin to talk about a post-pandemic scenario that demands as much attention as the initial crisis period. After the most critical moment of the health emergency has passed, we are left with social, economic, and emotional crises. Therefore, future studies can be conducted to assess whether psychological symptoms persisted 2 years after the COVID-19 pandemic among different segments of society, including survivors. It would also be important to compare the level of psychological impacts with the severity of the disease developed by infected individuals, determining whether these impacts were directly caused by the infection or its secondary consequences. Collectively, these studies can guide the development of public policies focused on the mental health damages caused by the pandemic.

### 4.1. Limitations

The present study’s main limitation was the data collection methodology. As this was an exclusively remote survey carried out during the COVID-19 pandemic, all information collected was self-reported by participants through electronic forms. Thus, it was not possible to test the participants to identify whether any subject in the control group, despite having reported no symptoms, was not infected with SARS-CoV-2.

## 5. Conclusion

Overall, this study found a higher prevalence of post-traumatic stress disorder, depression, anxiety, and insomnia in patients affected by COVID-19 when compared to uninfected ones. Despite this, the only statistically significant difference between the studied populations was in the levels of insomnia. In summary, the surviving population of SARS-CoV-2 virus infection tends to show little difference in terms of the development of PTSD, anxiety, and depression when compared to non-infected individuals. On the other hand, disorders such as insomnia are more prevalent and with a significant difference between the groups, appearing more in infected individuals.

## Data availability statement

The original contributions presented in the study are included in the article/supplementary material, further inquiries can be directed to the corresponding author.

## Ethics statement

The studies involving human participants were reviewed and approved by the Comitê de Ética em Pesquisa do Instituto de Ciências e Saúde da Universidade Federal do Pará. The patients/participants provided their written informed consent to participate in this study.

## Author contributions

OS: study conception and design. SS, LC, FB, RS, and OS: methodology and data collection. SS, LC, FB, and RS: modeling, statistical, and descriptive analysis. SS, LC, and FB: article edition. RS and OS: scientific consultants and correction supervision. All authors read and approved the final manuscript.

## Conflict of interest

The authors declare that the research was conducted in the absence of any commercial or financial relationships that could be construed as a potential conflict of interest.

## Publisher’s note

All claims expressed in this article are solely those of the authors and do not necessarily represent those of their affiliated organizations, or those of the publisher, the editors and the reviewers. Any product that may be evaluated in this article, or claim that may be made by its manufacturer, is not guaranteed or endorsed by the publisher.
